# Supplementary material for: Long-Term Protein Restriction Modulates Lipid Metabolism in White Adipose Tissues and Alters Colonic Microbiota of Shaziling Pigs
Source: Animals (Basel). 2022 Oct 26;12(21):2944. doi: 10.3390/ani12212944 (PMC9654241; doi:10.3390/ani12212944)
Supplement: Supplementary file 1 [file animals-12-02944-s001.zip › animals-1933039-supplementary 1.pdf]

C/EBPα

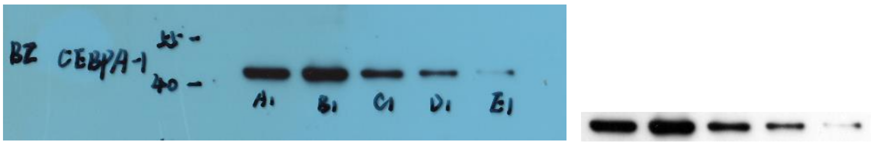

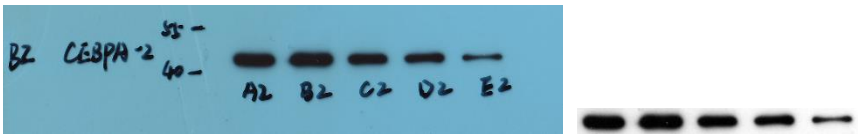

selected

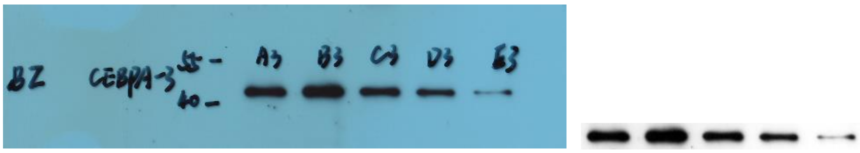

GAPDH

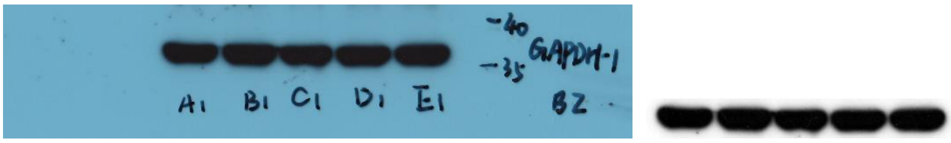

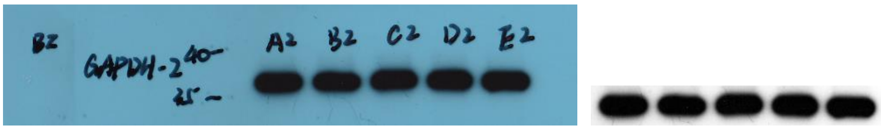

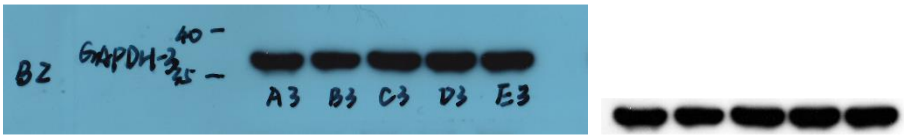

p-AMPK

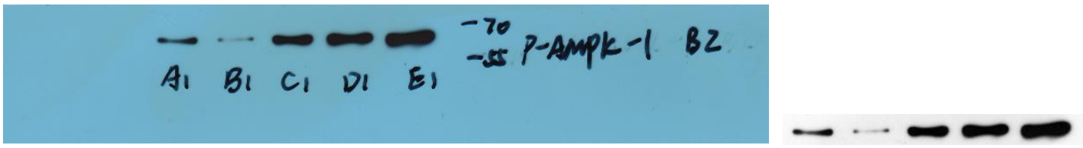

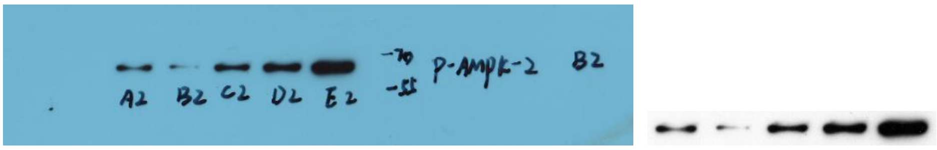

selected

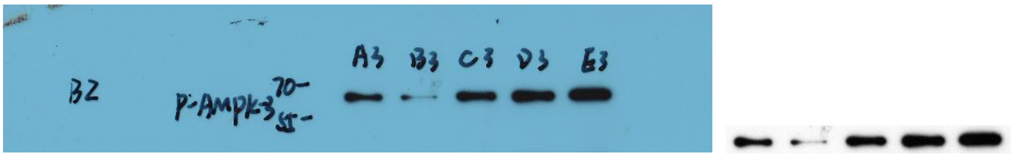

p-mTOR

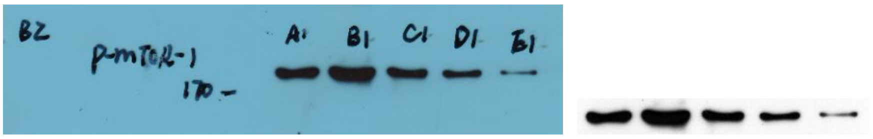

selected

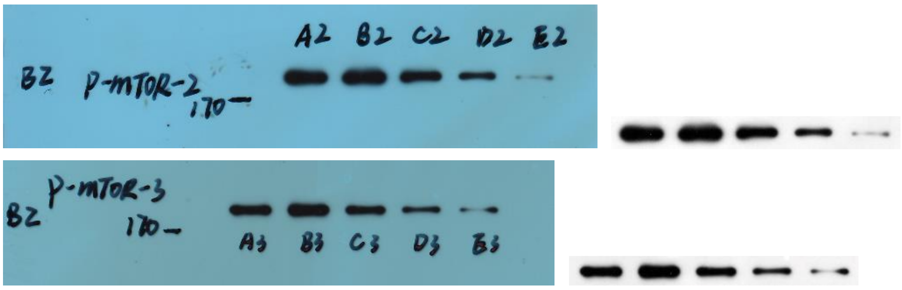

PPARγ

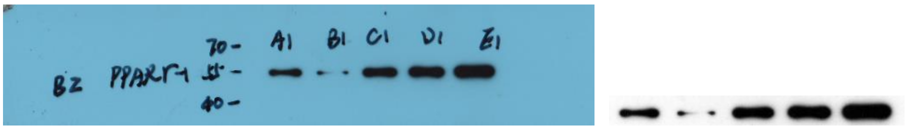

selected

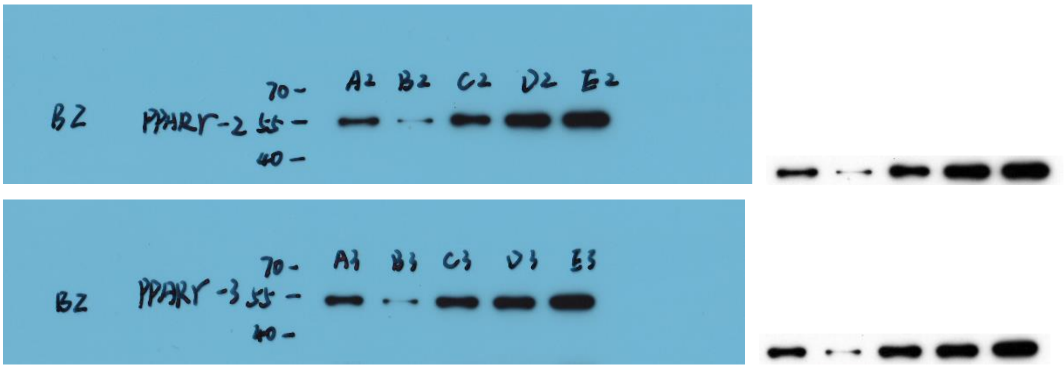

Figure S1: Western blot bands for perirenal adipose tissue

C/EBPα

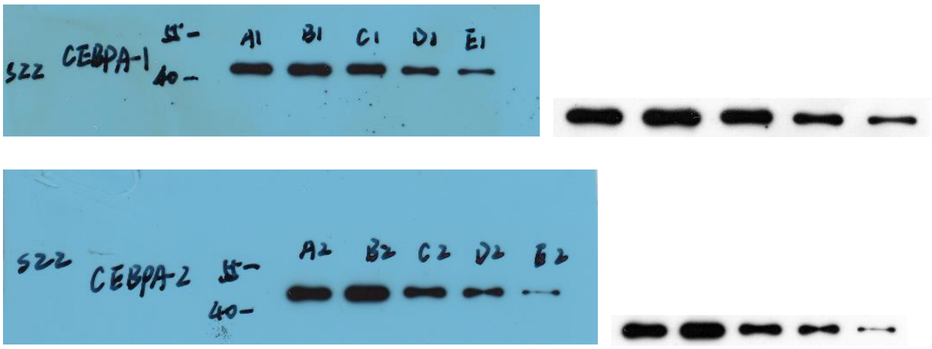

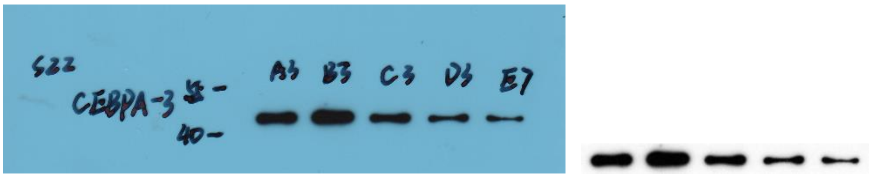

selected

GAPDH

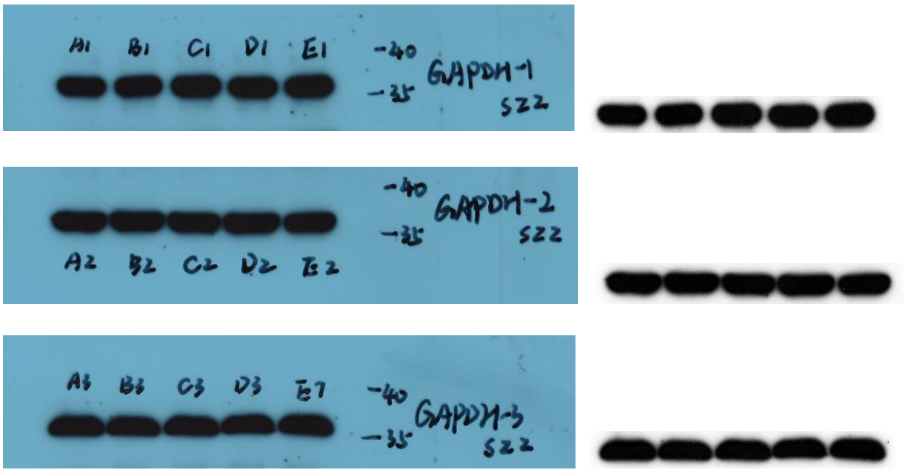

p-AMPK

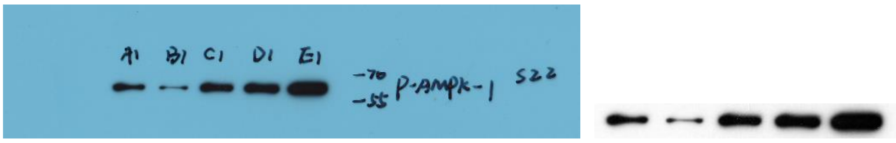

selected

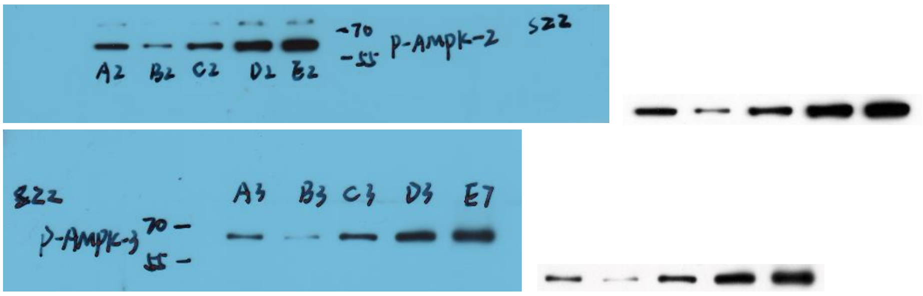

p-mTOR

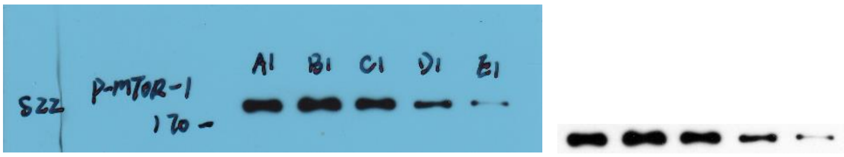

selected

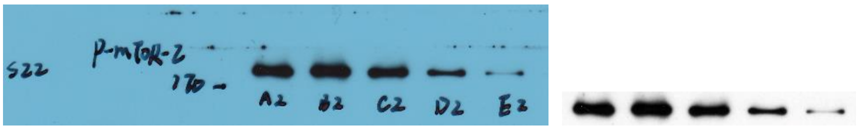

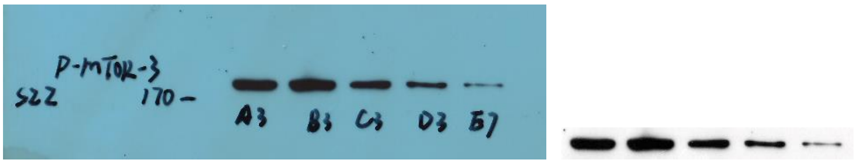

PPARγ

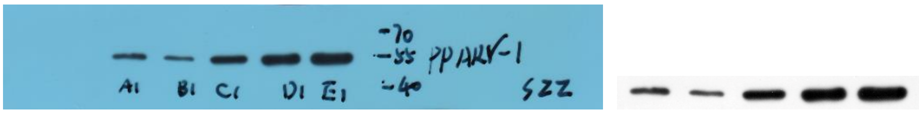

selected

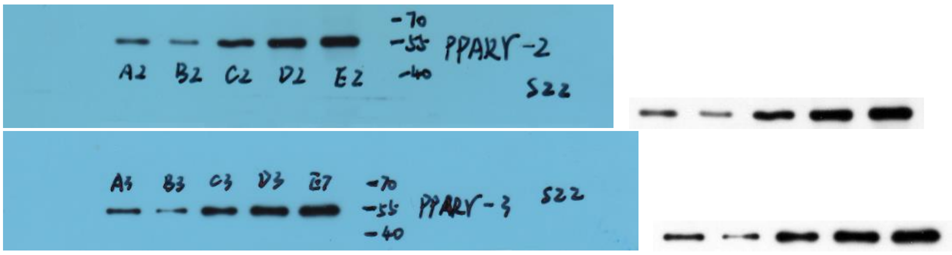

Figure S2: Western blot bands for dorsal subcutaneous adipose tissue
