# Supplementary material for: Long-Term Protein Restriction Modulates Lipid Metabolism in White Adipose Tissues and Alters Colonic Microbiota of Shaziling Pigs
Source: Animals (Basel). 2022 Oct 26;12(21):2944. doi: 10.3390/ani12212944 (PMC9654241; doi:10.3390/ani12212944)
Supplement: Supplementary file 1 [file animals-12-02944-s001.zip › animals-1933039-supplementary 2.pdf]

Category	Phase (kg)	Digestable energy (MJ/kg)	Crude protein (%)	Calcium (%)	Phosphorus (%)	Salt (%)
Replacement gilts	30~50	11.70	13	0.6	0.5	0.3
Pregnant sows	early stage	11.29	11	0.61	0.5	0.32
	late stage	11.70	13	0.61	0.5	0.32
Lactating sows	/	12.54	15	0.64	0.5	0.44
Breeding boars	/	12.54	15	0.66	0.5	0.35
Piglets	5~10	13.38	20	0.7	0.6	0.25
	10~15	13.38	18	0.65	0.55	0.25
	15~30	12.54	16	0.55	0.45	0.3
Growing-finishing pigs	30~50	12.12	14	0.55	0.45	0.3
	>50	12.70	12	0.5	0.4	0.3
